# Metabolic profiling of *Eurotium cristatum* during liquid fermentation using untargeted UHPLC–MS/MS metabolomics

**DOI:** 10.3389/fmicb.2026.1921106

**Published:** 2026-08-11

**Authors:** Chengyu Shi, Luyi Wang, Min Zhao, Zhi Wang, Luqi Yin, Wenjie Ning, Liqiang Wang

**Affiliations:** 1 School of Medicine, Huaqiao University, Quanzhou, China; 2 Department of Nuclear Medicine, No. 940 Hospital of Joint Logistics and Security Force, Chinese People’s Liberation Army, Lanzhou, Gansu, China; 3 Department of Special Medicine, Daping Hospital, Third Military Medical University, Chongqing, China

**Keywords:** differential metabolites, *Eurotium cristatum*, KEGG pathway, liquid fermentation, untargeted metabolomics

## Abstract

Untargeted metabolomics based on ultra-high-performance liquid chromatography–tandem mass spectrometry (UHPLC–MS/MS) was used to systematically characterize metabolites in the liquid fermentation broth of *Eurotium cristatum* at 0, 24, and 40 h. Principal component analysis (PCA), partial least squares discriminant analysis (PLS-DA), differential metabolite screening, metabolite classification, and KEGG pathway enrichment analysis were applied to investigate dynamic metabolic changes and key regulatory pathways during fermentation. PCA explained 82.20% of the total variance, clearly separating samples from different fermentation stages and revealing phased metabolic patterns. Amino acids, peptides and their analogues (29.03%), lipids and lipid-like molecules (17.06%), and organic acids (8.40%) were the predominant metabolite classes showing significant stage-dependent changes. The metabolite profiles suggested that early fermentation was mainly associated with primary metabolic processes supporting fungal growth, whereas the later fermentation stage was characterized by the increased abundance of metabolites associated with secondary metabolism, with substantial accumulation of putative bioactive metabolites, including TCA cycle-related organic acids, terpenoids, and lipids. Pathway analysis using the KEGG database showed that differentially expressed metabolites were primarily linked to metabolic pathways, amino acid metabolism, biosynthesis of secondary metabolites, nucleotide metabolism, and ABC transporter pathways. These results provide a foundation for industrial fermentation of *E. cristatum* and targeted production of high-value metabolites.

## 1 Introduction

*Eurotium cristatum* (*E. cristatum*) is the dominant filamentous fungus responsible for the characteristic fermentation of Fuzhuan brick tea with broad application potential, and widely used in the production of fermented foods, pharmaceuticals, and health supplements (Zhang et al., 2025). This fungus plays an important ecological role and has attracted increasing attention in food and pharmaceutical industries due to its unique bioactive properties (Yi et al., 2025). During liquid fermentation, *E. cristatum* is capable of synthesizing a variety of bioactive metabolites, such as amino acids, nucleotides, organic acids and terpenoids (Deng et al., 2024; Wang et al., 2022). These metabolites exhibit a wide range of biological activities, particularly antioxidant and antimicrobial effects (Chen et al., 2023; Fan et al., 2025), which contribute to their extensive applications in food preservation, nutritional enhancement, and disease prevention (Sun et al., 2025).

Previous investigations have primarily concentrated on elucidating the metabolic role of *E. cristatum* during the solid-state fermentation of black tea (Sun et al., 2023; Xu et al., 2025), research into the dynamic changes in metabolites and the conversion of key pathways at different fermentation stages in its liquid fermentation system remains relatively limited and requires further elucidation (Yi et al., 2025). Liquid fermentation is a key method for culturing *E. cristatum*, and the types and quantities of its metabolites vary across different fermentation stages, which are closely related to the metabolic patterns of the fungus, environmental conditions, and the composition of the culture medium (Huang et al., 2023; Xie et al., 2022). However, traditional metabolite analysis methods primarily rely on the detection of single targets (Qiu et al., 2025), making it difficult to comprehensively and systematically reveal the complex metabolic changes during fermentation (Xie et al., 2022; Yang C. et al., 2025). In recent years, with the rapid development of metabolomics technologies, particularly the widespread application of ultra-high-performance liquid chromatography–tandem mass spectrometry (UHPLC-MS/MS) (Zhao et al., 2026; Zhou et al., 2022), untargeted metabolomics has become one of the key techniques for elucidating changes in microbial metabolites (Lu et al., 2022). This technology not only identifies and analyses a vast number of metabolites but also provides information on the dynamic changes in metabolic networks during fermentation (Elsheshtawy et al., 2026; Liu et al., 2025).

In our preliminary experiments, the growth characteristics of *E. cristatum* were evaluated by monitoring the reducing sugar content during fermentation. The reducing sugar concentration decreased from 11.77 to 2.14 g/L after 40 h of fermentation, indicating that the carbon source had been largely consumed and that fungal growth and metabolite production had approached a stable phase. Therefore, 40 h was selected as the late fermentation time point for metabolomic analysis, together with 0 h and 24 h, to capture dynamic metabolic changes throughout the fermentation process.

Despite increasing interest in the metabolites produced by *E. cristatum*, the temporal metabolic remodeling that occurs during liquid fermentation remains insufficiently understood. In particular, the dynamic changes in metabolite composition and the associated metabolic pathways at different fermentation stages have not been comprehensively characterized. Therefore, this study employed untargeted UHPLC–MS/MS metabolomics (Carrillo et al., 2022; Liao et al., 2023) to systematically investigate the metabolite profiles of *E. cristatum* during liquid fermentation at 0, 24, and 40 h. By integrating multivariate statistical analysis, differential metabolites screening, chemical classification, and KEGG pathway enrichment analysis, we aimed to characterize stage-dependent metabolic alterations and provide new insights into metabolite accumulation during liquid fermentation, thereby providing a theoretical basis for process optimization and targeted production of valuable metabolites.

## 2 Materials and methods

### 2.1 Chemicals, fungal strain, and equipment

*Eurotium cristatum* (AK-97) was obtained from Yuyuan Tea Industry Limited Company, (Quanzhou, Fujian, china). Methanol and acetonitrile (Fisher Chemical, HPLC, China); Water (Fisher Chemical, LC-MS, China); ammonia solution and formic acid (CNW Technologies, HPLC, China); isopropanol (Merck, HPLC, China); 2-Chloro-L-phenylalanine (analytical, Adamas-beta, China); Vanquish Horizon UHPLC System, Exploris 240 Mass Spectrometer (Thermo Scientific, USA).

### 2.2 Preparation of *E. cristatum* fermentation broth and sample extraction

Fermentation medium contained 7.5% sucrose, 1.5% yeast extract, 0.5% magnesium sulfate, and 0.1% dipotassium hydrogen phosphate, autoclaved at 121 °C for 20 min. Under aseptic conditions, a starter culture was prepared using Potato Dextrose Broth (PDB) at a seeding rate of 2.5%. The medium and starter were mixed and transferred to a fermenter for liquid fermentation at 28 °C, pH 5.5, 30% dissolved oxygen, and 180 rpm for 40 h. Samples collected at 0, 24, and 40 h were designated as FJY0h, FJY24h, and FJY40h, and four biological replicates. Mycelium was collected and freeze-dried for 72 h.

Metabolites were extracted from broth samples by adding 400 μL of methanol–acetonitrile solution (1:1, v/v) containing L-2-chlorophenylalanine (0.02 mg/mL) as an internal standard to 200 μL of sample. After vortex mixing for 30 s, the samples were subjected to ultrasonication (40 kHz, 5 °C) for 30 min, followed by incubation at −20 °C for 30 min. The extracts were then centrifuged at 13,000 × *g* for 15 min at 4 °C, and the supernatant was collected and dried under nitrogen gas. The dried sample was resuspended in 120 μL of acetonitrile–water (1:1, v/v), vortexed, ultrasonicated for 5 min, and centrifuged again, and transferred to a sample vial for analysis. Quality control (QC) samples were prepared by pooling equal aliquots (20 μL) of the supernatants from all analyzed samples. During the UHPLC-MS/MS analysis, QC samples were inserted at regular intervals, with one QC sample analyzed after every 5–15 study samples.

### 2.3 Chromatographic and mass spectrometry conditions

Metabolomic profiling was conducted using a Thermo UHPLC–Exploris 240 mass spectrometry platform. Chromatographic separation was achieved on an ACQUITY UPLC HSS T3 column (100 mm × 2.1 mm, 1.8 μm; Waters, Milford, MA, USA). The mobile phase consisted of solvent A, comprising 95% water and 5% acetonitrile supplemented with 0.1% formic acid, and solvent B, composed of 47.5% acetonitrile, 47.5% isopropanol, and 5% water containing 0.1% formic acid. The column temperature was maintained at 40 °C, and 3 μL of each sample was injected for analysis. Elution was performed at a flow rate of 0.35 mL/min using the following gradient program: 2% B from 0 to 1 min, increased to 5% B from 1 to 2 min, linearly ramped from 5% to 95% B between 2 and 8 min, returned to 5% B from 8 to 9 min, and held at 5% B until 10 min.

Electrospray ionization (ESI) in positive and negative modes; scan range m/z 70–1050; sheath gas 60 arb, auxiliary gas 20 arb; oven temperature 350 °C capillary 320 °C, spray voltage 3400 V (positive) and −3000 V (negative); S-Lens 70; collision energies 20%, 40%, 60%; Full MS resolution 60,000; MS^2^ resolution 15,000.

### 2.4 Data analysis

Metabolomic data were preprocessed with Progenesis QI v3.0 (Waters) through a workflow comprising baseline correction, feature extraction, peak area integration, retention time alignment, and cross-sample feature matching (Wang et al., 2025; Yang X. et al., 2025). Metabolite characterization was carried out through MS/MS spectral comparisons with entries in the HMDB and Metlin databases, together with an internal reference database. Metabolites exhibiting significant differences between groups were selected according to the following criteria: VIP > 1 from the PLS-DA model, |log_2_FC| > 1, and *p* < 0.05.

## 3 Results and analysis

### 3.1 Total ion chromatograms

**FIGURE 1:**
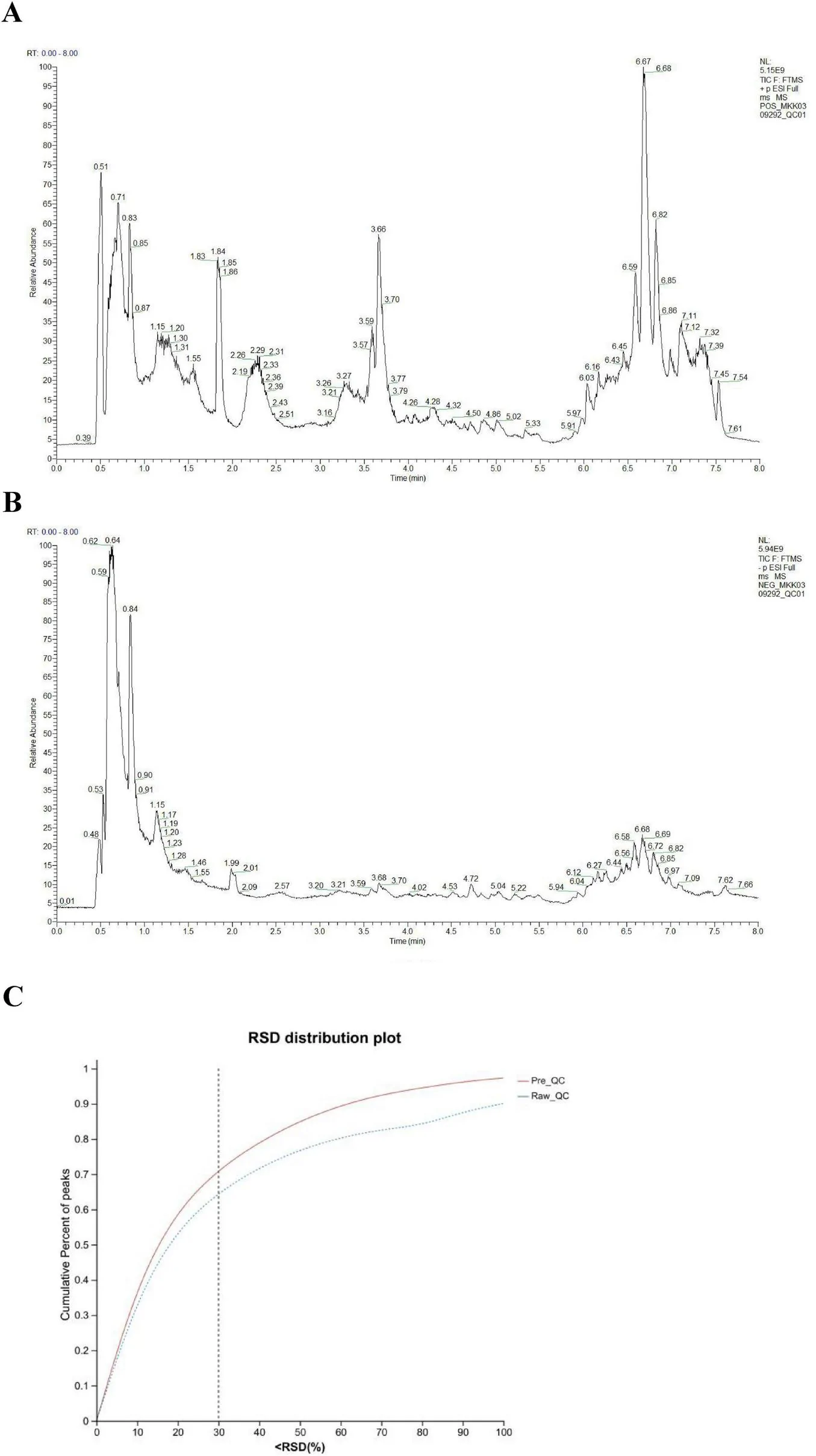
Quality assessment of UHPLC-MS/MS metabolomics data. **(A)** Total ion chromatograms (TICs) of QC samples in positive ion mode (ESI+). **(B)** Total ion chromatograms (TICs) of QC samples in negative ion mode (ESI–). **(C)** RSD distribution of detected metabolic features in QC samples.

Under the analytical conditions employed, the chromatographic peaks exhibited good shape and uniform distribution, with stable ion responses throughout the analysis (Figures 1A, B). The reproducibility and stability of the metabolomic dataset were further evaluated based on the RSD distribution of detected features in QC samples (Figure 1C), approximately 70% of the detected metabolic features exhibited RSD values below 30% after data preprocessing, indicating satisfactory analytical repeatability and stability of the UHPLC-MS/MS platform. These results demonstrate that the acquired metabolomic data were reliable and suitable for subsequent multivariate statistical analysis and differential metabolite screening.

### 3.2 Results of PCA and sample correlation analysis

**FIGURE 2:**
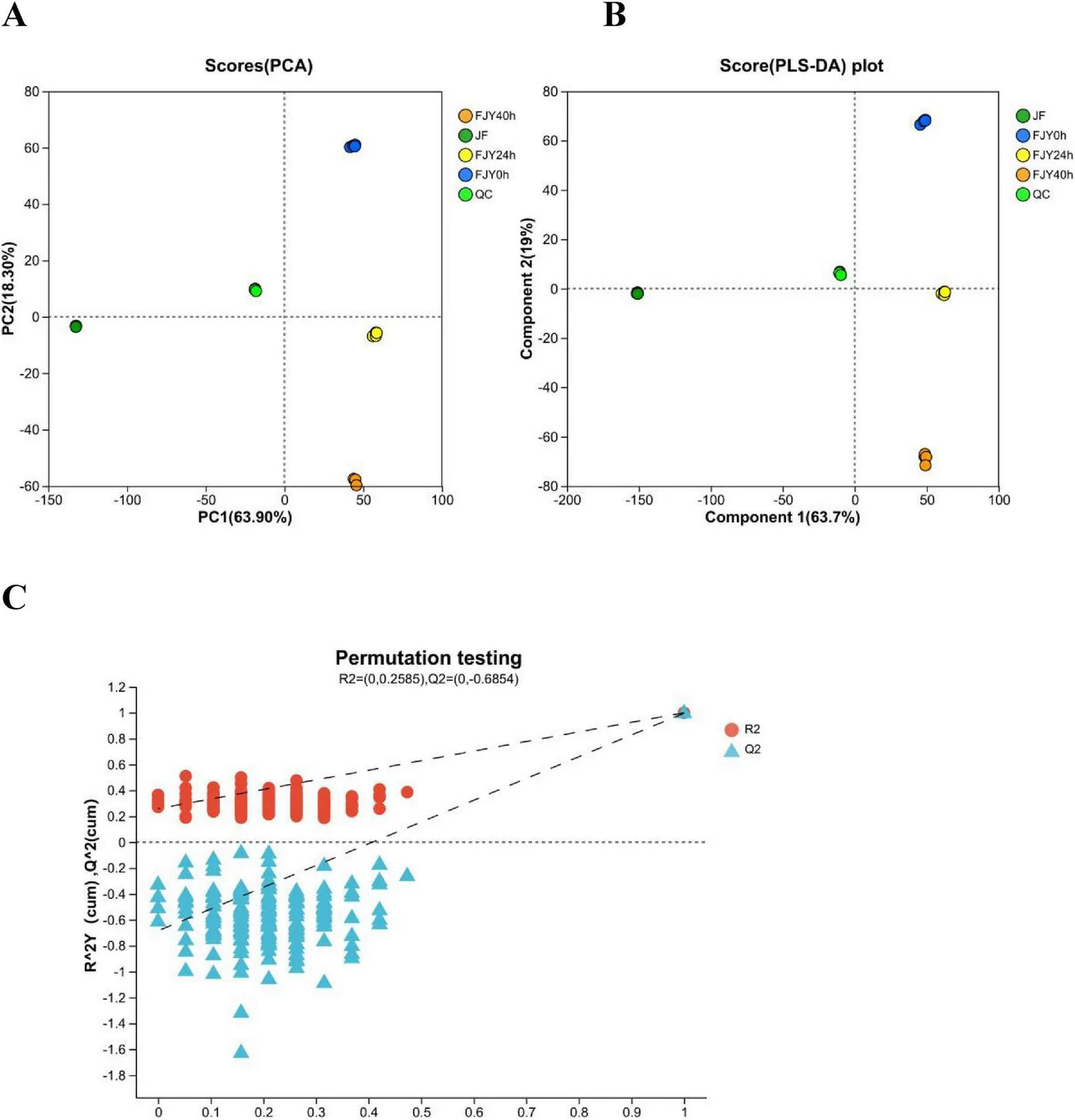
Multivariate statistical analysis of metabolites during liquid fermentation of *E. cristatum*. **(A)** Principal component analysis (PCA) score plot. **(B)** Partial least squares-discriminant analysis (PLS-DA) score plot. **(C)** Permutation test validating the robustness of the PLS-DA model.

Principal component analysis revealed PC1 and PC2 accounted for 63.90% and 18.30% of the total variance, respectively, resulting in a cumulative variance contribution of 82.20%. These results effectively reflected the overall variation among samples (Figure 2A). Samples from different fermentation stages were clearly separated, whereas samples within the same group exhibited good clustering. Component 1 and Component 2 explained 63.7% and 19.0% of the total variance, respectively, indicating good explanatory power of the model (Figure 2B). These findings suggest significant differences in metabolite composition among fermentation stages and demonstrate strong discrimination between sample groups.

The permutation test results for the PLS-DA model showed that the original model exhibited an R^2^ value of 0.2585 and a Q^2^ value of −0.6854 both of which were higher than those obtained from permutation testing, whereas the intercept of the Q^2^ regression line was negative (Figure 2C). These results indicate that the model was not overfitted and possessed satisfactory predictive ability and stability, confirming the reliability of the analysis.

### 3.3 Differential metabolite screening and distribution characteristics

**FIGURE 3:**
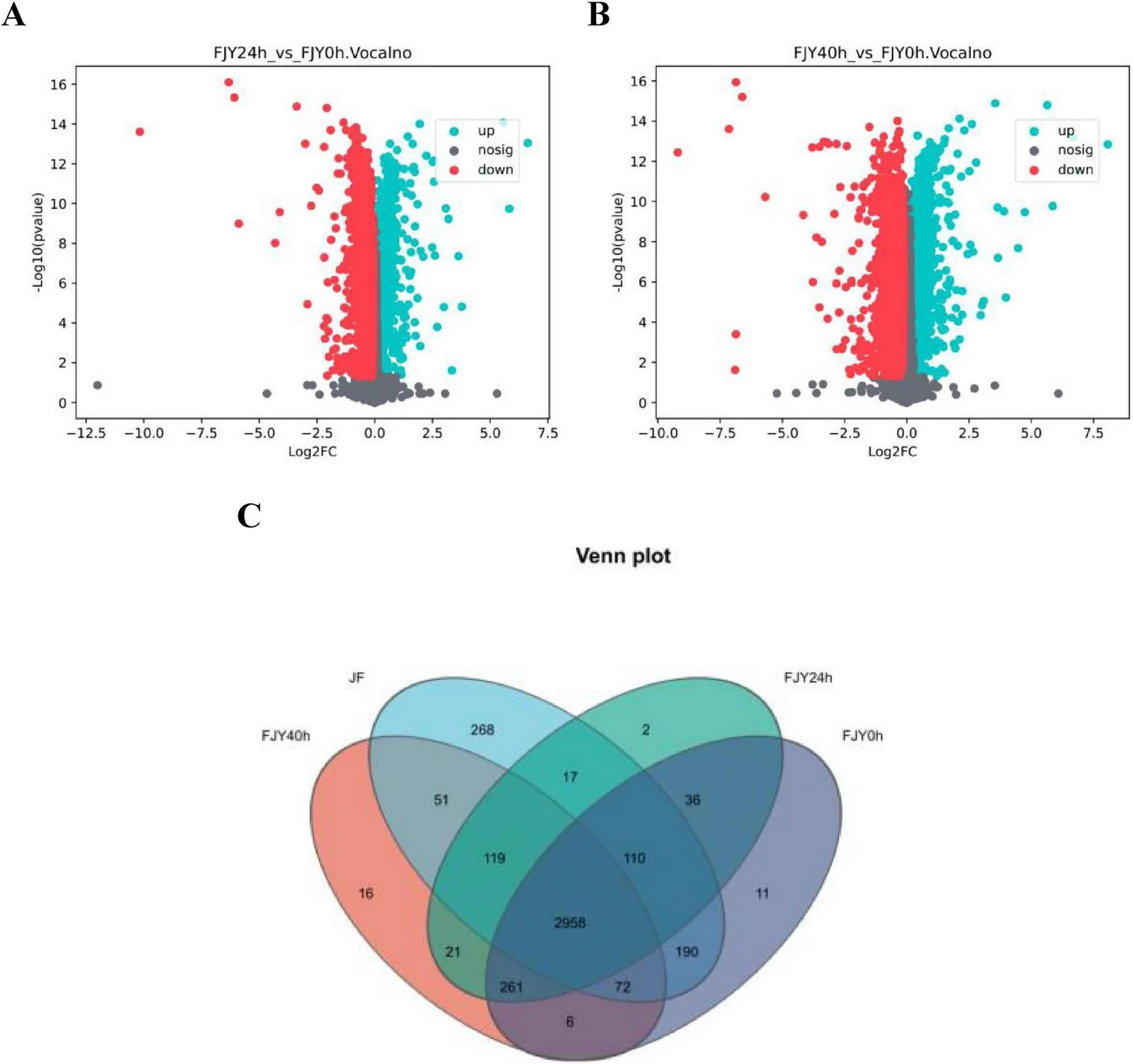
Identification and distribution of differential metabolites during liquid fermentation of *E. cristatum*. **(A)** Volcano plot of FJY24h versus FJY0h. **(B)** Volcano plot of FJY40h versus FJY0h. **(C)** Venn diagram showing shared and stage-specific differential metabolites among fermentation stages.

Compared with FJY0h, 75 metabolites were significantly upregulated and 89 were significantly downregulated in FJY24h (Figure 3A). As fermentation progressed to 40 h, the number of differential metabolites increased markedly, reaching 122 upregulated and 195 downregulated metabolites (Figure 3B). In both comparisons, downregulated metabolites predominated over upregulated metabolites. These results indicate that metabolic perturbations became progressively more pronounced with fermentation time, reflecting enhanced metabolite utilization and metabolic reprogramming during the later stage of liquid fermentation.

Venn diagram analysis was conducted to evaluate the distribution of differential metabolites among fermentation stages (Figure 3C). The identification of 2,958 metabolites shared across all fermentation samples suggests the existence of a core metabolome maintained throughout fermentation. In addition, each fermentation stage contained unique differential metabolites. The number of stage-specific metabolites was highest in the late fermentation stage (FJY40h), indicating more extensive metabolic regulation during this period.

### 3.4 Cluster analysis of differentially expressed metabolites and characteristics of key metabolite changes

**FIGURE 4:**
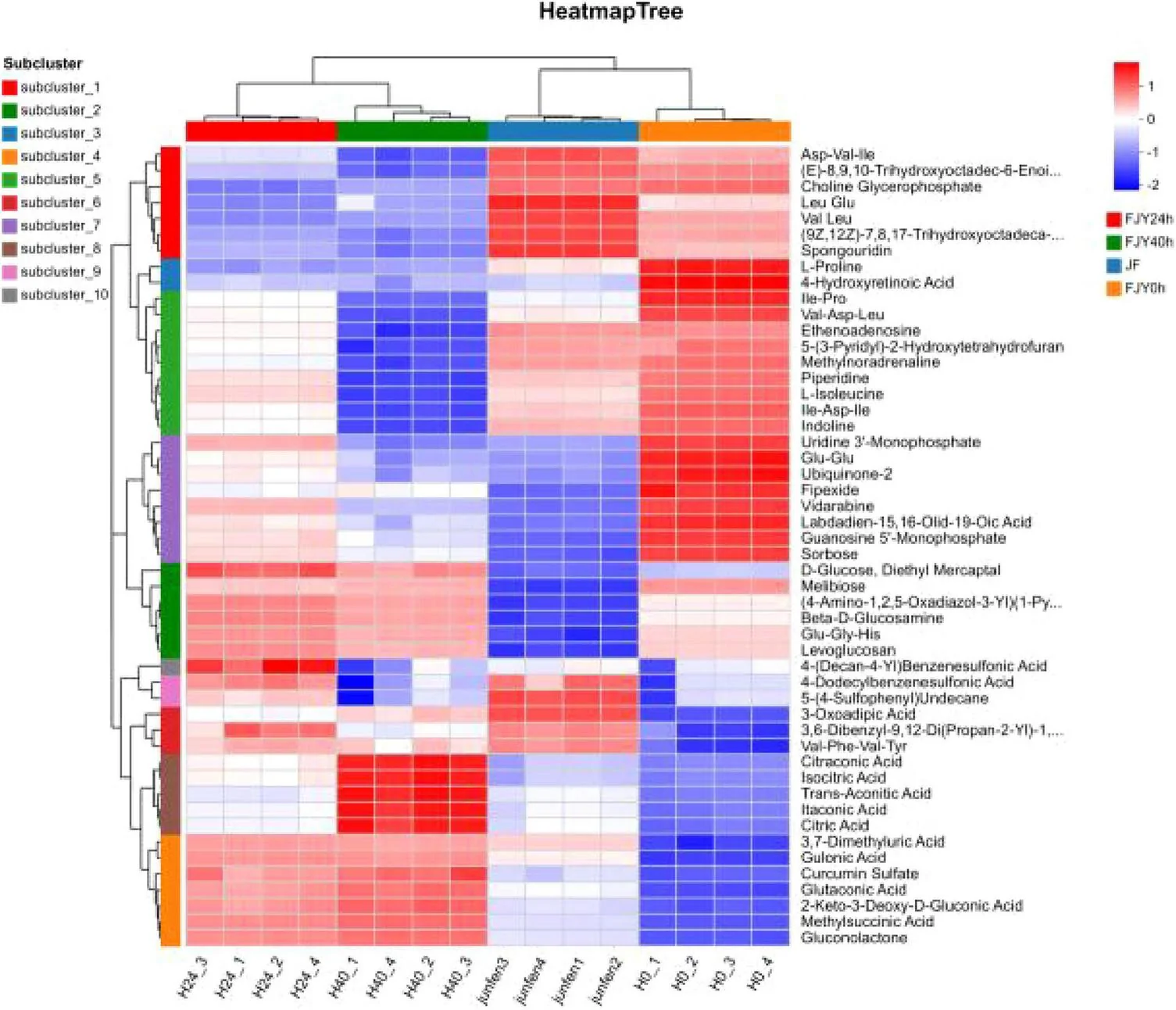
Hierarchical clustering heatmap of differential metabolites detected at different fermentation stages of *E. cristatum*.

Hierarchical clustering analysis in Figure 4. Samples from different fermentation stages exhibited distinct clustering patterns, biological replicates within the same stage clustered closely together, whereas samples from different stages were clearly separated. These results indicate that the metabolic profile of *E. cristatum* changed systematically during liquid fermentation.

At the start of fermentation (FJY0h), the abundances of various amino acids (L-proline, L-isoleucine, Asp-Ile, glutamic acid) and nucleotides (uridine and guanosine), as well as ubiquinone 2 and sorbitol, were relatively high. This suggests that cell growth and basal metabolism were active at this stage, and the accumulation of amino acid and nucleotide may be associated with membrane synthesis, energy requirements and nucleic acid synthesis. In addition, elevated levels of glutamic acid and sorbitol indicate active nitrogen metabolism and carbon source utilization. During the middle stage of fermentation (FJY24h), the abundance of benzenesulfonic acid, methylsuccinic acid, glutaric acid, citric acid and gluconolactone increased. These changes suggest continued carbon and nitrogen metabolism accompanied by enhanced organic acid biosynthesis. Increased levels of glutamate and methylsuccinate may be associated with amino acid synthesis, energy metabolism, and organic acid production. During the late fermentation stage (FJY40h), metabolites including glutamic acid, methylsuccinic acid, gluconolactone, citraconic acid, citric acid, isocitric acid, trans-aconitic acid, and itaconic acid further accumulated. These changes indicate enhanced secondary metabolism. In particular, the increased abundance of citric acid, isocitric acid, and trans-aconitic acid suggests a shift in metabolic activity toward energy metabolism and secondary metabolite production.

### 3.5 Analysis of the chemical classification characteristics of differential metabolites

**FIGURE 5:**
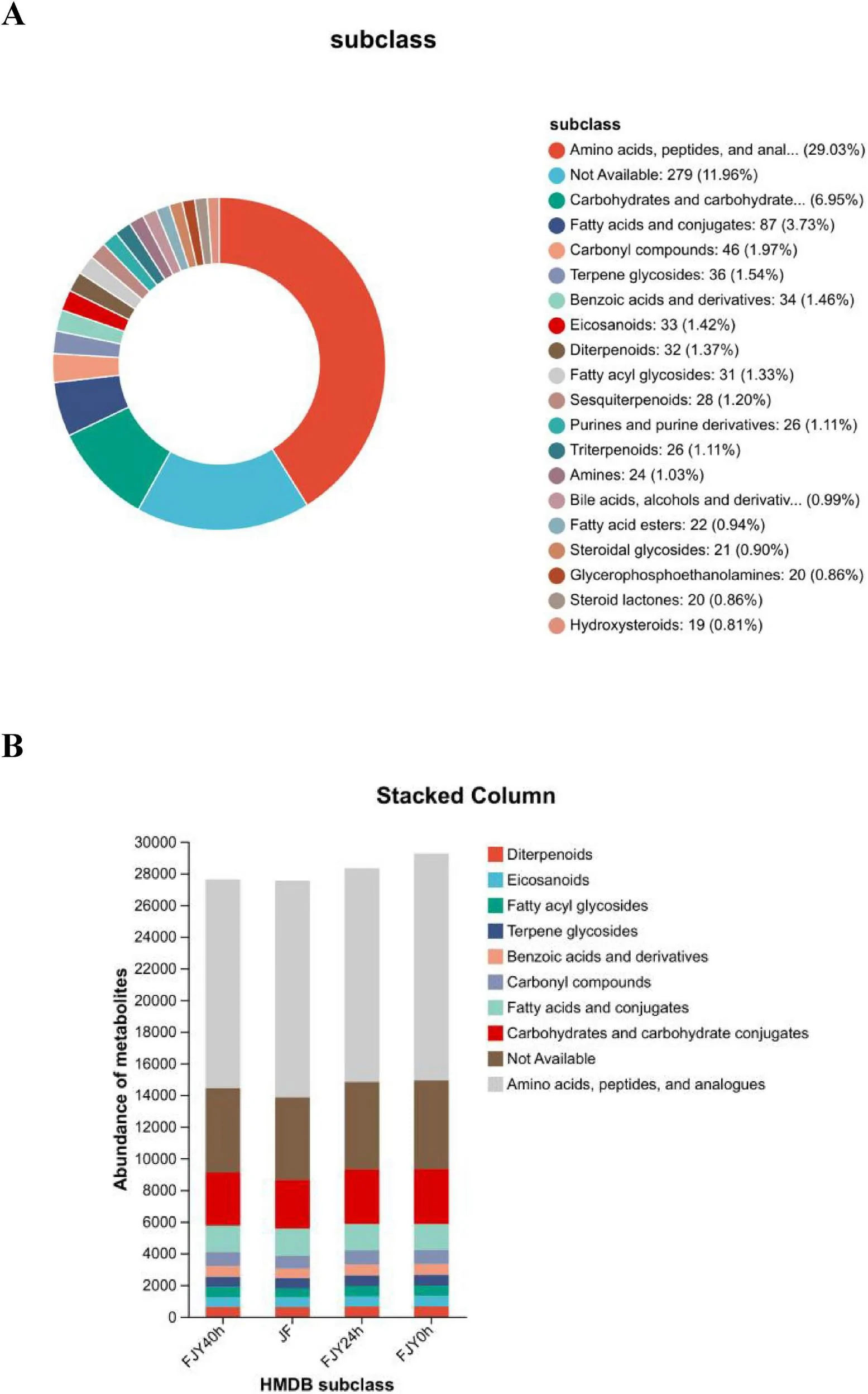
Chemical classification and temporal variation of differential metabolites during liquid fermentation of *E. cristatum*. **(A)** Relative distribution of different chemical classes of differential metabolites. **(B)** Dynamic changes in the abundance of major metabolite classes across fermentation stages.

Based on the identified differentially expressed metabolites, chemical classification was performed using HMDB and KEGG databases (Figure 5A). During the fermentation process, differential metabolites were mainly distributed among amino acids, peptides and their analogues (29.03%), lipids and lipid-like molecules (17.06%), other organic acids (8.40%), purines and purine derivatives (1.11%). Amino acid-related metabolites represented the most abundant group of differential compounds, indicating the central role of amino acid metabolism during fermentation. Furthermore, organic acids and lipid metabolites also contributed substantially, participating in energy metabolism and maintaining cellular homeostasis.

The temporal changes in major metabolite classes are shown in Figure 5B. At the early fermentation stage (FJY0h), growth and basal metabolism were primarily supported by amino acids, peptides and derivatives, along with fatty acids and derivatives, with particularly high levels of fatty acid glycerides. During mid-fermentation (FJY24h), the abundances of fatty acids, their derivatives, and diterpenoid metabolites increased significantly, alongside a gradual increase in organic acids such as benzoic acid derivatives. These changes indicate a shift in carbon metabolism and enhanced organic acid synthesis. In the late fermentation stage (FJY40h), metabolite abundances further increased, with fatty acids, their derivatives, and diterpenoid metabolites reaching peak levels, marking the predominance of secondary metabolite synthesis.

### 3.6 KEGG pathway enrichment analysis

**FIGURE 6:**
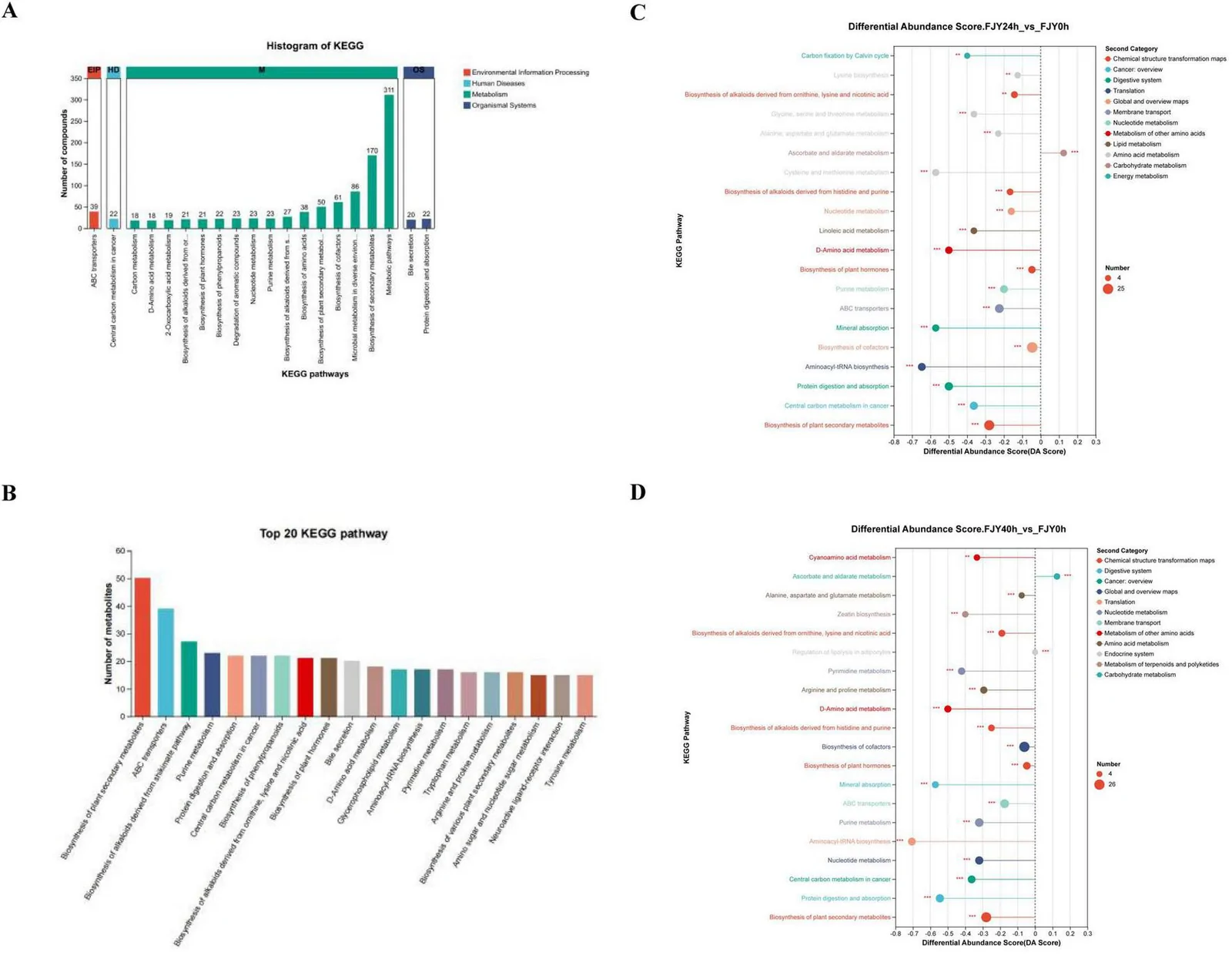
KEGG pathway enrichment analysis of differential metabolites during liquid fermentation of *E. cristatum*. **(A)** KEGG pathway classification of differential metabolites. **(B)** Top 20 significantly enriched KEGG pathways. **(C)** Differential abundance (DA) analysis of KEGG pathways in FJY24h versus FJY0h. **(D)** Differential abundance (DA) analysis of KEGG pathways in FJY40h versus FJY0h.

The KEGG pathway classification of differential metabolites is shown in Figures 6A, B. The differential metabolites were predominantly enriched in pathways related to primary metabolism, secondary metabolite biosynthesis, ABC transporters, nucleotide metabolism, and amino acid metabolism. Analysis of the top 20 KEGG pathways highlighted “Biosynthesis of plant secondary metabolites” as the most significantly enriched pathway during fermentation, consistent with the elevated levels of secondary metabolites, including diterpenoids and organic acids in the later stages.

Differential abundance (DA) analysis of KEGG pathways was performed (Figures 6C, D). A positive DA score indicates more upregulated metabolites, whereas a negative DA score indicates predominance of downregulated metabolites. Between FJY24h and FJY0h, metabolites involved in cysteine and methionine metabolism, amino acid metabolism, aminoacyl-tRNA biosynthesis, biosynthesis of plant secondary metabolites, the Calvin cycle, and ABC transporter pathways generally showed decreased abundance compared with FJY0h. The metabolites involved in these pathways, primarily consisting of organic acids, acid-derived compounds, including glutamic acid, succinic acid, cysteine, and valine, provide energy for cell proliferation, support protein synthesis, and maintain basic cellular functions, while also serving as precursors for secondary metabolites such as citric acid, isocitric acid, and trans-aconitic acid.

Between FJY40h and FJY0h, metabolites associated with pyrimidine metabolism, purine metabolism, nucleotide metabolism, and aminoacyl-tRNA biosynthesis generally showed lower abundance in FJY40h. Differential metabolites in these pathways included citric acid, isocitric acid, alkaloids, terpenoids, purines, pyrimidines, and nucleotides. These results suggest that in the late fermentation stage, cells enter a resource-conserving phase with reduced demand for amino acids, minerals, and nucleotides, while focusing on the synthesis of organic acids and secondary metabolites. The decreased abundance of nucleotide, pyrimidine, and purine metabolism indicates slowed nucleic acid synthesis and energy consumption, likely associated with reduced cell proliferation and transition to metabolic stability.

### 3.7 Metabolite correlation and metabolic network analysis

**FIGURE 7:**
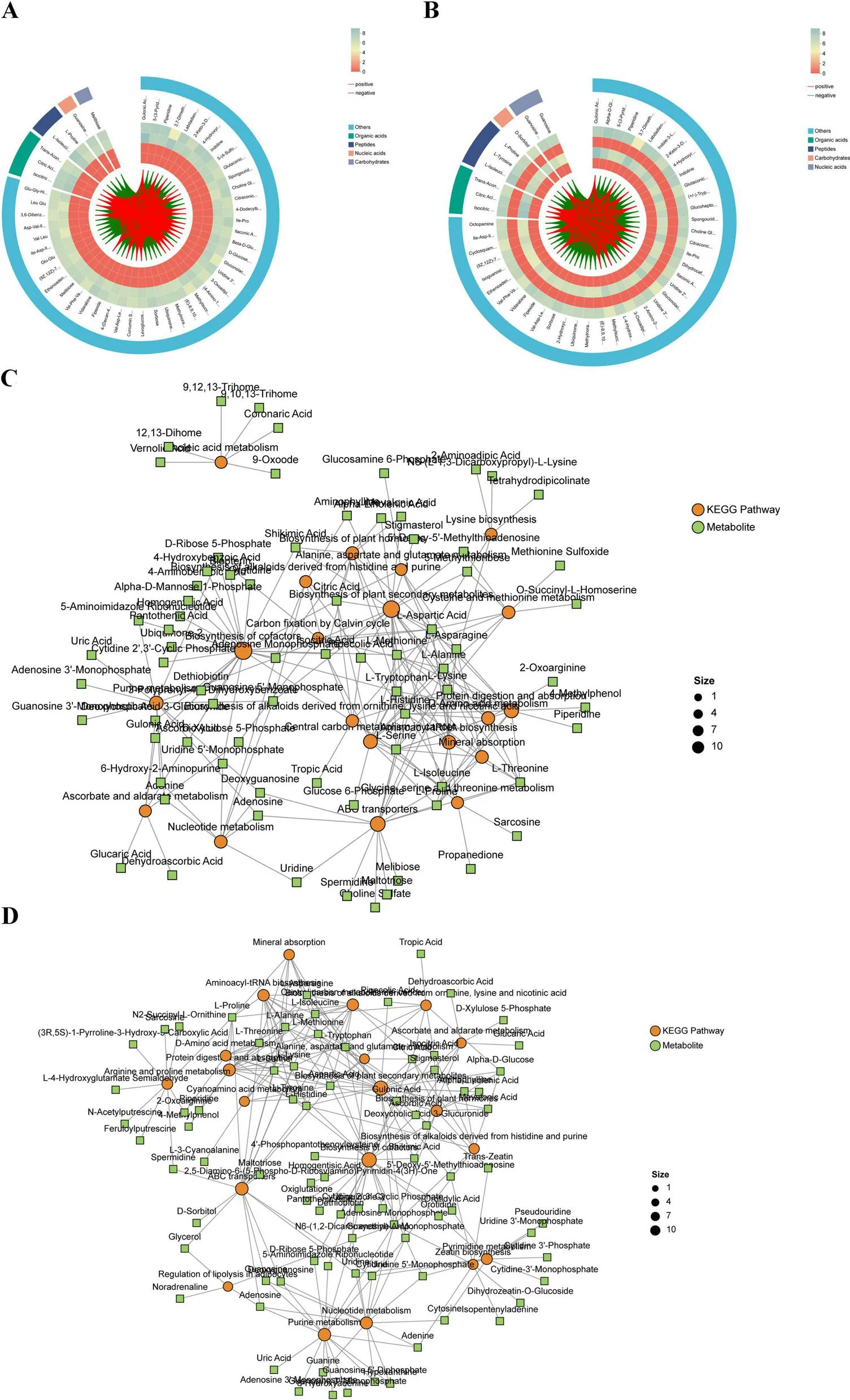
Correlation and network analysis of differential metabolites during liquid fermentation of *E. cristatum*. **(A)** Correlation chord diagram for FJY24h versus FJY0h. **(B)** Correlation chord diagram for FJY40h versus FJY0h. **(C)** Metabolic interaction network of differential metabolites in FJY24h versus FJY0h. **(D)** Metabolic interaction network of differential metabolites in FJY40h versus FJY0h.

Changes in relative metabolite abundances during fermentation were analyzed using correlation chord diagrams (Figures 7A, B). During the mid-fermentation stage, positively correlated metabolites included amino acids and their derivatives (e.g., proline, isoleucine, glutamic acid), organic acids (e.g., citric acid), secondary metabolites, and sugars (e.g., gluconolactone), which showed marked increases in abundance. The upregulation of these metabolites indicates enhanced energy metabolism, antioxidant capacity, and anti-inflammatory potential during the mid-fermentation stage, with vigorous carbon source metabolism supporting fungal growth. Conversely, negatively correlated compounds such as leucine, adenosine, adenine arabinoside, and sorbitol decreased in abundance, suggesting a shift in carbon and nitrogen utilization toward organic acid biosynthesis.

In the late fermentation stage, positively correlated metabolites, including amino acids such as valine and methionine, continued to increase, indicating ongoing protein synthesis and nitrogen metabolism to balance energy demands. Significant accumulation of organic acids, including sorbitol, citric acid, isocitric acid, trans-aconitic acid, methylsuccinic acid, gluconolactone, itaconic acid, and citraconate, reflects a metabolic shift toward energy storage and secondary metabolite production, with enhanced antioxidant and defense-related activities. Negatively correlated metabolites, including guanosine, guanosine-5′-monophosphate, proline, tyrosine, and isoleucine, decreased in abundance, indicating reduced amino acid metabolism and nucleic acid synthesis. The downregulation of nucleosides including guanosine, adenosine, and adenine arabinoside suggests diminished nucleic acid demand in later stages, reduced cell proliferation, and a metabolic focus on energy conservation and secondary metabolite accumulation.

The differential metabolites network (Figures 7C, D) revealed significant enrichment of multiple pathways during mid-fermentation, including plant secondary metabolism, cofactor biosynthesis, ABC transporters, and protein digestion and absorption. Key metabolites involved included citric acid, serine, aspartic acid, isocitric acid, shikimic acid, and adenosine monophosphate. These results indicate that during mid-fermentation, metabolism shifts toward amino acid synthesis, energy metabolism, and antioxidant responses, with increased requirements for nitrogen and energy. Cellular growth and metabolic activity were active, with enhanced protein and organic acid biosynthesis.

In the late fermentation stage, pathways including plant secondary metabolism, cofactor biosynthesis, ABC transporters, protein digestion and absorption, and nucleotide metabolism were significantly enriched. The metabolic focus shifted toward antioxidant defense, energy metabolism, and secondary metabolite biosynthesis. Key metabolites included ascorbic acid, linolenic acid, D-glucose, isocitric acid, aspartic acid, tyrosine, histidine, lysine, tryptophan, alanine, isoleucine and threonine. These results indicate a progressive enhancement of antioxidant defense and fatty acid metabolism during the late fermentation stage.

## 4 Conclusions and discussion

Untargeted metabolomics revealed distinct stage-dependent changes in the metabolite composition of *E. cristatum* during liquid fermentation. At 0 h, basal growth metabolism predominated, with high abundances of primary metabolites including amino acids, nucleotides, and carbohydrates. At 24 h, the metabolic transition phase was evident, with active carbon and nitrogen metabolism and gradual accumulation of organic acids and lipids. By 40 h, metabolites associated with secondary metabolism accumulated to higher levels, significant accumulation of organic acids involved in the tricarboxylic acid (TCA) cycle, such as citric acid, isocitric acid, trans-aconitic acid, and itaconic acid, was accompanied by elevated levels of putative bioactive compounds with antioxidant and antibacterial activities. These results provide a theoretical basis for optimizing industrial fermentation processes and for the targeted production of valuable metabolites from *E. cristatum*.

Itaconic acid, citric acid, and isocitric acid, in addition to their roles as TCA cycle intermediates, have been reported to possess antioxidant, anti-inflammatory, and antibacterial activities (Alhawiti, 2024). Solvent extraction studies have shown that antioxidant components of *E. cristatum* are mainly concentrated in ethyl acetate fractions, likely related to phenolic and flavonoid compounds. Furthermore, fermentation broth of *E. cristatum* has been shown to enhance immune function in immunosuppressed mice (Zhang et al., 2024). The current metabolomics results are generally consistent with previous observations, demonstrating that accumulation of organic acids, terpenoids, and lipid metabolites during late fermentation may provide the material basis for antioxidant and immunomodulatory activities. In addition, enhanced accumulation of TCA cycle intermediates may reflect enhanced central carbon metabolism and increased availability of precursor metabolites for secondary metabolite biosynthesis. In particular, the accumulation of citric acid, isocitric acid, trans-aconitic acid, and itaconic acid may reflect remodeling of carbon metabolism during the late fermentation stage, which may facilitate the biosynthesis of putative bioactive compounds and may contribute to metabolic adaptation of *E. cristatum* under nutrient-limited conditions.

Comparison with liquid fermentation of other *Aspergillus* species (Liao et al., 2023; Sun et al., 2025, 2023) indicates that *E. cristatum* also follows a primary-to-secondary metabolism transition. However, the relatively higher proportion of organic acids, especially itaconic acid and trans-aconitic acid, in the late stage suggests that *E. cristatum* possesses distinct metabolic advantages in terms of secondary metabolite diversity and organic acid accumulation, potentially reflecting its ecological adaptation as a probiotic in Fu Brick Tea.

The decreased abundance of metabolites associated with nucleotide, pyrimidine, and purine metabolism in late fermentation may be associated with metabolic reprogramming during late fermentation, may suggest changes in metabolic resource allocation during late fermentation, accompanied by increased accumulation of organic acids and other metabolites associated with secondary metabolism. Concurrent enrichment of ABC transporter pathways indicates enhanced metabolite efflux and defense responses in later stages, complementing the accumulation of secondary metabolites. Nevertheless, in this study, fermentation parameters, including temperature, pH, dissolved oxygen, and agitation speed, were maintained under controlled conditions to characterize the intrinsic metabolic dynamics of *E. cristatum* during liquid fermentation. However, environmental factors may influence metabolic regulation, and different fermentation conditions could potentially alter carbon utilization, stress responses, and secondary metabolite biosynthesis. Therefore, future studies combining metabolomics with fermentation parameter optimization and multi-omics approaches are needed to further elucidate the regulatory mechanisms underlying environmental adaptation. And untargeted metabolomics may yield false positives and is semi-quantitative. The biological functions of some differentially expressed metabolites require further validation through targeted metabolomics or *in vitro* assays. Additionally, molecular regulatory mechanisms underlying these metabolic changes, such as the expression of key enzyme genes, remain to be elucidated. Future studies integrating transcriptomics or proteomics could clarify the regulatory networks controlling the transition from primary to secondary metabolism and verify key metabolic nodes using gene knockout or overexpression approaches.

## Data Availability

The raw data supporting the conclusions of this article will be made available by the authors, without undue reservation.
